# Study on Damage Accumulation and Life Prediction with Loads below Fatigue Limit Based on a Modified Nonlinear Model

**DOI:** 10.3390/ma11112298

**Published:** 2018-11-16

**Authors:** Junhong Zhang, Xi Fu, Jiewei Lin, Zhiyuan Liu, Nuohao Liu, Bin Wu

**Affiliations:** 1 State Key Laboratory of Engines, Tianjin University, Tianjin 300072, China; zhangjh@tju.edu.cn (J.Z.); liuzhiyuan940304@163.com (Z.L.); knowhow07@163.com (N.L.); 18340828871@163.com (B.W.); 2 Renai College, Tianjin University, Tianjin 301636, China

**Keywords:** nonlinear damage accumulation, loads below fatigue limit, life prediction, strengthening effect, compressor blade

## Abstract

Most fatigue theories neglect the loads below fatigue limit in damage accumulation, which leads to inconsistency between the predicted and the actual fatigue lives. In this study, a novel damage model is proposed to take into account the loads below fatigue limit from two aspects: the strengthening effect and the cumulative damage. The strengthening effect is introduced by an exponential function and the cumulative damage is calculated by fuzzy method with membership functions (MFs). The proposed model is verified against the experimental data under variable amplitude loading conditions. It is found the modified model with Cauchy MF significantly reduces the relative error of predicted life from 35.18% (linear model) and 16.09% (original Chaboche model) to 8.38% (proposed model). As a case study, the proposed damage model is implemented to evaluate the service life of a compressor blade under variable amplitude loading spectrum containing small loads below the fatigue limit.

## 1. Introduction

Over the past few decades of fatigue damage research, it is found that damage accumulation depends on loading parameters, such as the stress ratio, the mean stress and the loading sequence [1]. So the Miner’s linear damage rule [2] is inevitable to miscalculate fatigue life due to the assumption that the cumulative damage per cycle is independent of load. Nonlinear damage rules [3,4,5,6,7,8] are proposed to correlate the damage accumulation with loading parameters. Dierkes [9] pointed out that the key of those rules is the calculation of the exponent, which is precisely the problem restricting the model application. Since the essence of fatigue rupture is a process of energy loss, damage models with definite physical meaning are established from the energy point of view [10,11] to reveal the evolution of damage accumulation. Sosnovskiy [12] analysed the concept of entropy in continuum mechanics and thermodynamics to study the tribo-fatigue and a nonlinear damage model is developed regarding the damage accumulation as an irreversible process [13,14,15]. These sorts of damage model consider the correlation between loading parameters and cumulative damage [16,17].

In most cases, damage models do not matter the load below the fatigue limit of material. It means the life would be infinite if only loads below fatigue limit is applied. In engineering, it is very common that the loading history contains low-amplitude loads lower than the fatigue limit, sometimes this kind of low-amplitude load even takes the majority. Under this condition, the low/high-amplitude interaction may change the damage accumulation process [18,19], and the low-amplitude load may cause considerable damage as well. Therefore, life prediction and damage assessment would be inaccurate without considering the effect of the low-amplitude load below the fatigue limit. Sinclair [20] presented a concept of coaxing phenomenon to describe the improvement in fatigue resistance due to the understressing. It is indicated that the fatigue life could be extended by low-amplitude loads below the fatigue limit [21,22]. Lu [23,24] found that the strengthening effect relates to the stress range. For some materials the strengthening effect is obvious under constant low-amplitude loading but weakened under variable amplitude loading [25]. The material is still possible to fail during the low-amplitude loading process [26]. In order to study the influence of the strengthening effect on damage behaviour, Zhu [27] proposed a modified linear rule on the basis of the S–N curve so the loads below the fatigue limit cannot be calculated. Hence, the cumulative damage only can be calculated according to a hypothetical reference value from the fatigue test. However, load parameters of engineering structure vary continuously with operation conditions. Therefore, a considerable amount of low-amplitude load below the fatigue limit may exist in the varying cyclic fatigue load, which would bring notable difference in life prediction. As a result, it is necessary to refine the damage model to take into account the influence of low-amplitude loads below fatigue limit. Meanwhile, some other important loading parameters (e.g., the loading sequence, the variation of load amplitude) of significant influences on the fatigue damage accumulation should be involved as well.

In this paper, a modified damage model is proposed to consider the effect caused by the low-amplitude loading cycles below the fatigue limit on the damage accumulation. The low-amplitude load effect is described using a strengthening function and the fuzzy method. Comparisons between experimental and predicted fatigue lives from various damage models are conducted. After the model validation, the fatigue life of an aero-engine compressor blade is evaluated using the actual loading spectrum.

## 2. Damage Model

### 2.1. Nonlinear Damage Accumulation

The fatigue behaviour of material can be regarded as a continuum process that the damage continuously accumulates until failure occurs. On this basis, Chaboche and Lesne [28,29] established the differential relationship between the damage variable, *D*, and the loading cycle, *n*:

(1) $$dD={\left[1-{\left(1-D\right)}^{\beta +1}\right]}^{\alpha }{\left[\frac{\sigma_{a}}{M_{0}\left(1-b_{0}\sigma_{m}\right)\left(1-D\right)}\right]}^{\beta }dn\;$$

where *σ*_a_ is the stress amplitude, *σ*_m_ is the mean stress, and *α*, *β*, *M*_0_, and *b*_0_ are material parameters which can be determined by fatigue test [17]. Equation (1) can be rewritten into a generalised form:

(2) $$\frac{dD}{dn}=f^{-1}\left(\sigma_{a},\sigma_{m},D\right)\cdot g\left(\sigma_{a},\sigma_{m}\right)\;$$

where *f*(·) and *g*(·) are functions of loading parameters and damage derived from Equation (1). Letting the initial damage be *D*_0_ and the critical damage be *D*_c_, one can obtain:

(3) $$\forall D\in \left[D_{0},D_{c}\right],\exists \int_{D_{0}}^{D_{c}}f\left(\sigma_{a},\sigma_{m},D\right)dD=ng\left(\sigma_{a},\sigma_{m}\right)\;$$

**Figure 1:**
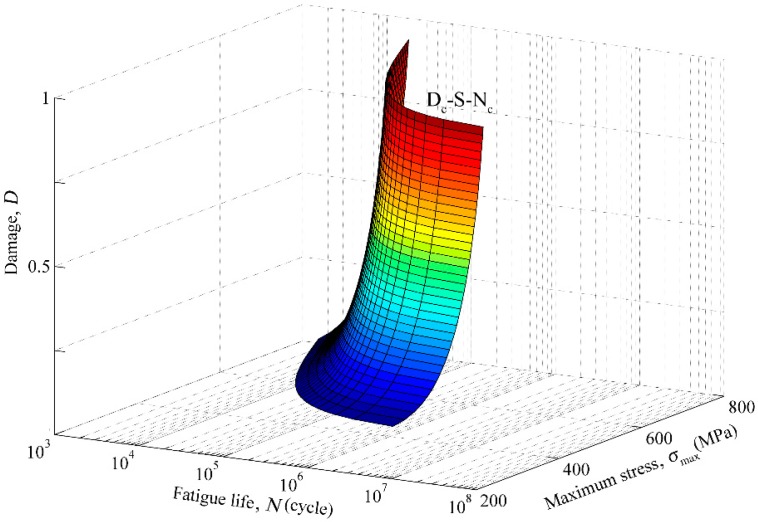
D–S–N surface defined by the Chaboche model.

Equation (3) describes the nonlinearity between loads, damage and loading cycles in the whole life process (see Figure 1). Integrating Equation (1) from *D* = 0 (brand new condition without any damage) to *D* = 1 (fractured), the fatigue life and the damage after *n* loading cycles can be obtained [28]:

(4) $$N_{f}=\frac{1}{1-\alpha }\frac{1}{1+\beta }{\left[\frac{M_{0}\left(1-b_{0}\sigma_{m}\right)}{\sigma_{a}}\right]}^{\beta }\;$$

(5) $$D=1-{\left[1-{\left(\frac{n}{N_{f}}\right)}^{\frac{1}{1-\alpha }}\right]}^{\frac{1}{1+\beta }}\;$$

### 2.2. Effect of Load Below Fatigue Limit

**Figure 2:**
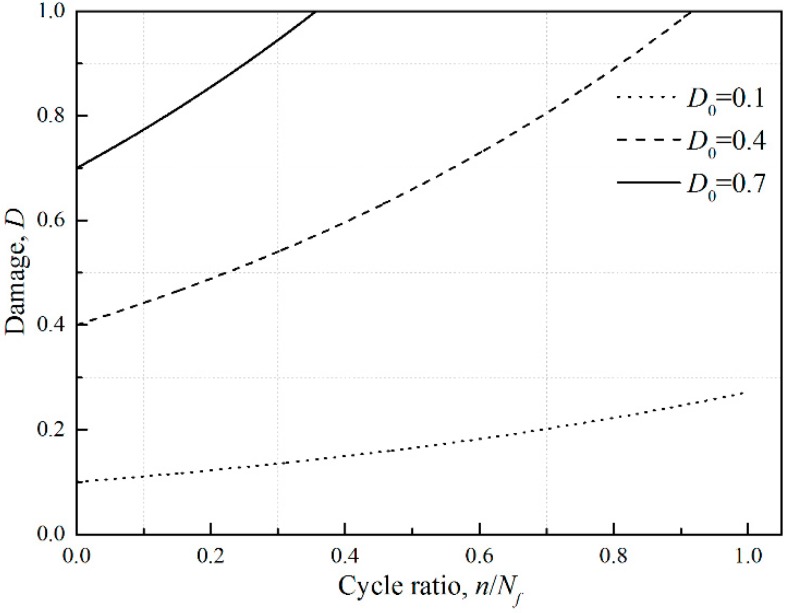
Damage curves under loads below fatigue limit with different initial damages.

Damage caused by loads below the fatigue limit is described as following [28]:

(6) $$N_{i}^{*}={\left[\frac{M_{0}\left(1-b_{0}\sigma_{m}\right)}{\sigma_{a}}\right]}^{\beta }\;$$

(7) $$D_{i}=D_{i-1}\exp\left(\frac{n_{i}}{N_{i}^{*}}\right)\;$$

where N_i_* is the fatigue cycle of the *i*th low-amplitude load and *D_i_* is the corresponding damage parameters. For constant amplitude loading, *D_i_*_−1_ equals zero and Equation (7) retrogresses back to the traditional fatigue method that the below-fatigue limit load does not cause any damage. But Chaboche model thinks that the load below the fatigue limit will still introduce damage accumulation if initial damage exists. With different initial damages of 0.1, 0.4, and 0.7, the damage accumulation curves under loads below fatigue limit are shown in Figure 2.

It is shown that the damage accumulation rate increases with increasing initial damage under the same cycle ratio. The strength degeneration could be responsible for this phenomena: a higher initial damage means a less residual life and the deteriorated material cannot even afford loads under fatigue limit. 

### 2.3. Proposed Model

**Figure 3:**
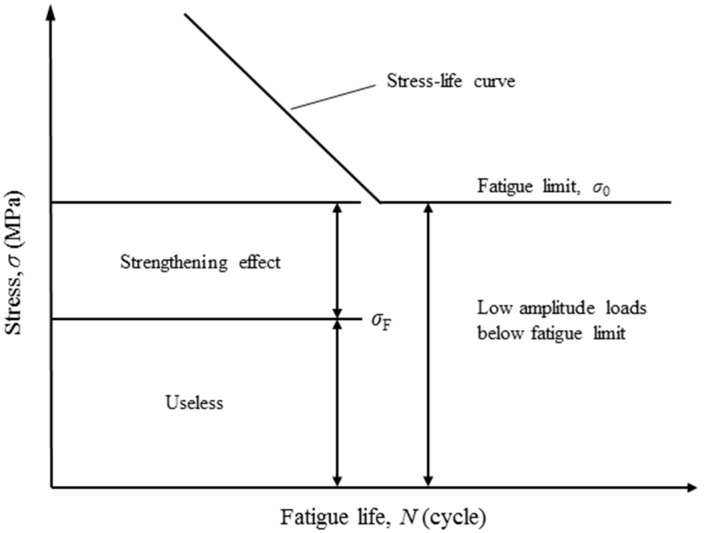
Low-amplitude loads below fatigue limit [27].

In Chaboche model, all low-amplitude loads are considered contributing to the fatigue damage accumulation. However, it has been stated that under the repeated low-amplitude loads the fatigue strength of the metallic material can be improved to some extents (so called the strengthening effect) [20,23,24]. As shown in Figure 3, the loads higher than the fatigue limit result in damage accumulation with no doubts, while those below the fatigue limit should be further identified. Some of the low-amplitude loads can affect the damage accumulation (e.g., the strengthening effect) and the others could be useless. Note that the low-amplitude load with strengthening effect still produces damage at the same time. In life prediction, therefore, methods omitting the low-amplitude loads would give overestimation, while methods (e.g., Chaboche model) counting all the low-amplitude loads would give underestimation. On this basis, a strengthening function, *f*_s_, is employed to describe the low-amplitude load effect reasonably:

(8) $$f_{s}=\{\begin{array}{c} 1\\ \exp\left(m\prime \sigma_{i}\right) \end{array}\begin{array}{c} \;\sigma_{i}\in [0,\sigma_{F}]\\ \;\sigma_{i}\in [\sigma_{F},\sigma_{0}] \end{array}\;$$

where *m*’ is the strengthening coefficient related to material properties and can be obtained by test, and *σ*_F_ is the lower limit of the strengthening stress.

For *σ* < *σ*_F_, the strengthening function equals 1, which means the load is too small to perform neither strengthening effect nor damage. For *σ*_F_ < *σ* < *σ*_0_, the strengthening function would be active so as to extend the fatigue life. Based on the experimental results [24], the range of the low-amplitude load with strengthening effects is determined around 75% to 95% of the fatigue limit of material. Therefore, the lower boundary of the strengthening load is defined as *σ*_F_ = *λσ*_0_ (*λ* = 0.75–0.95). Then the fatigue life under the specific loading condition of *σ*_F_ < *σ_i_* < *σ*_0_ can be calculated as follows:

(9) $$N_{i}^{*}{}^{\prime }=\exp\left(m\sigma_{i}\right)\cdot {\left[\frac{M_{0}\left(1-b_{0}\sigma_{m}\right)}{\sigma_{a}}\right]}^{\beta }\;$$

Meanwhile the damage under the same low-amplitude load is:

(10) $$D_{i}=\exp(m\sigma_{i})D_{i-1}\exp\left(\frac{n_{i}}{N_{i}^{*\prime }}\right)\;$$

In the variable amplitude loading condition, the model still can consider the strengthening effect caused by the low-amplitude loads. In a two-level loading case, the structure is firstly subjected to a stress *σ*_1_ for *n*_1_ cycles and then another stress *σ*_2_ for *n*_2_ cycles to failure. Assuming that the fatigue life for *σ*_1_ is *N*_1_, the damage *D*_1_ can be obtained:

(11) $$D_{1}=1-{\left[1-{\left(\frac{n_{1}}{N_{1}}\right)}^{\frac{1}{1-\alpha_{1}}}\right]}^{\frac{1}{1+\beta }}\;$$

For stress *σ*_2_, defining an equivalent cycle number *n*_2_*’* that can result the same amount of damage as *n*_1_ cycles at *σ*_1_, the damage accumulation can be rewritten as:

(12) $$D=1-{\left[1-{\left(\frac{n_{1}}{N_{1}^{}}\right)}^{\frac{1}{1-\alpha_{1}}}\right]}^{\frac{1}{1+\beta }}=1-{\left[1-{\left(\frac{n_{2}^{\prime }}{N_{2}^{}}\right)}^{\frac{1}{1-\alpha_{2}}}\right]}^{\frac{1}{1+\beta }}\;$$

And *n*_2_ can be calculated by the following expression:

(13) $$\frac{n_{2}}{N_{2}}=1-\frac{n_{2}^{\prime }}{N_{2}}=1-{\left(\frac{n_{1}}{N_{1}}\right)}^{\frac{1-\alpha_{2}}{1-\alpha_{1}}}\;$$

For a multi-level loading case, a cyclic ratio *Y_i_* is introduced [28]:

(14) $$Y_{i}=\frac{n_{i}^{\prime }+n_{i}}{N_{i}}={\left(D_{i}^{*}\right)}^{1-\alpha_{i}}\;$$

where *n_i_*‘ is the equivalent cycle number of the first (*i* − 1)th-level loads when the *i*th-level load is applied, *n_i_* is the loading cycles of the *i*th-level load, *N_i_*‘ is the fatigue life regarding the *i*th-level load and D_i_* is the substitution variable of damage *D_i_*. After *n*_1_ cycles under the first-level load *σ*_1_, *Y*_1_ and *D*_1_* can be expressed as follows:

(15) $$Y_{1}=\frac{n_{1}}{N_{1}}={\left(D_{1}^{*}\right)}^{1-\alpha_{1}}\;$$

(16) $$D_{1}^{*}={\left(\frac{n_{1}}{N_{1}}\right)}^{\frac{1}{1-\alpha_{1}}}\;$$

After *n*_2_ cycles under the second-level load *σ*_2_, *Y*_2_ and *D*_2_* can be expressed as:

(17) $$Y_{2}=\frac{n_{2}^{\prime }+n_{2}}{N_{2}^{}}={\left(\frac{n_{1}}{N_{1}^{}}\right)}^{\frac{1-\alpha_{2}}{1-\alpha_{1}}}+\frac{n_{2}}{N_{2}^{}}={\left(D_{1}^{*}\right)}^{1-\alpha_{2}}+\frac{n_{2}}{N_{2}^{}}={\left(D_{2}^{*}\right)}^{1-\alpha_{2}}\;$$

(18) $$D_{2}^{*}={\left[{\left(D_{1}^{*}\right)}^{1-\alpha_{2}}+\frac{n_{2}}{N_{2}^{}}\right]}^{\frac{1}{1-\alpha_{2}}}\;$$

Similarly, after *n_i_* cycles of the *i*th-level load *σ_i_*, *Y_i_* and D_i_* can be expressed as:

(19) $$Y_{i}=\frac{n_{i}^{\prime }+n_{i}}{N_{i}^{}}={\left(\frac{n_{i-1}^{\prime }+n_{i-1}}{N_{i-1}^{}}\right)}^{\frac{1-\alpha_{i}}{1-\alpha_{i-1}}}+\frac{n_{i}}{N_{i}^{}}={\left(D_{i-1}^{*}\right)}^{1-\alpha_{i}}+\frac{n_{i}}{N_{i}^{}}={\left(D_{i}^{*}\right)}^{1-\alpha_{i}}\;$$

(20) $$D_{i}^{*}={\left[{\left(D_{i-1}^{*}\right)}^{1-\alpha_{i}}+\frac{n_{i}}{N_{i}^{}}\right]}^{\frac{1}{1-\alpha_{i}}}\;$$

With the first *i*th-level loads that higher than the fatigue limit, the cumulative damage under the (*i* + 1)th-level load lower than the fatigue limit can be calculated by Equation (10), one can obtain:

(21) $$D_{i+1}^{*}=\exp(m\sigma_{i+1})D_{i}^{*}\exp\left(\frac{n_{i+1}}{N_{i+1}^{*\prime }}\right)\;$$

Then the damage accumulation for the (*i* + 2)th-level load above the fatigue limit can be given as:

(22) $$D_{i+2}^{*}={\left[{\left(D_{i+1}^{*}\right)}^{1-\alpha_{i+2}}+\frac{n_{i+2}}{N_{i+2}^{}}\right]}^{\frac{1}{{}^{1-\alpha_{i}+2}}}={\left[{\left(\exp(m\sigma_{i+1})D_{i}^{*}\exp\left(\frac{n_{i+1}}{N_{i+1}^{*\prime }}\right)\right)}^{1-\alpha_{i+2}}+\frac{n_{i+2}}{N_{i+2}^{}}\right]}^{\frac{1}{{}^{1-\alpha_{i+2}}}}\;$$

According to Equation (22), the fatigue life under multi-level variable amplitude loading with low-amplitude load strengthening effect can be obtained when the failure happens at $D_{i+2}^{*}=1$. But this model yet cannot determine whether the low-amplitude loads can cause damage or the strengthening effect. Therefore, in this study, membership functions (MFs) in the fuzzy mathematics theory are employed to help deciding which low-amplitude load should be included in damage accumulation [30,31]. In the ‘fuzzy region’ close to the fatigue limit, it is not necessary to obtain an absolute conclusion of causing damage or not, but only to describe how much the degree of the damage is [32,33]. The applicability of fuzzy set theory can be extended to the situations involving subjective uncertainty, or when the data are insufficient for statistical calculation. A fuzzy variable can be defined by MFs for describing the indeterminacy of damage. In the full load range from 0 to the maximum stress *σ*_max_, the MF $\mu_{\tilde{D}}\left(\sigma_{i}\right)$ can be defined as:

(23) $$\mu_{\tilde{D}}(\sigma_{i})=\{\begin{array}{ll} f_{\mathrm{fu}zzy}(\sigma_{i}) & \sigma_{i}\in [0,\sigma_{0})\\ 1 & \sigma_{i}\in [\sigma_{0},\sigma_{\max}] \end{array}\;$$

Substituting Equation (23) into Equation (22), we can get:

(24) $$D_{i+2}^{*\prime }={\left[{\left(\exp(m\sigma_{i+1})D_{i}^{*}\exp\left(\frac{n_{i+1}}{N_{i+1}^{*\prime }}\right)\right)}^{1-\alpha_{i+2}}\mu_{\tilde{D}}(\sigma_{i})+\frac{n_{i+2}}{N_{i+2}^{}}\right]}^{\frac{1}{{}^{1-\alpha_{i+2}}}}\;$$

where $0\leq \mu_{\tilde{D}}\left(\sigma_{i}\right)\leq 1$. Comparing with the Chaboche model and the Miner’s rule, the damage accumulation calculated by Equation (24) considers the strengthening effect of low-amplitude loads, of which the indeterminacy of damage degradation is determined by using fuzzy set theory. As the given capability, the proposed model can consider the effects of load sequence and nonlinearity on the damage accumulation.

The fuzziness is quantitatively described by the MF, so it is possible to exhibit the degree of fuzzification more definitely in application. In Equation (23), *μ_D_*(*σ_i_*) is a monotonous augment function between 0 and 1. For *σ > σ*_0_, *μ_D_*(*σ_i_*) is always equal to 1, because the damage caused by the load above the fatigue limit can be calculated definitely. For *σ < σ*_F_, the load can be omitted so that the value of MF is zero.

Distribution functions are generally used to describe the approximately fuzzy variable. Based on the previous studies [27,34,35,36], MFs frequently used are given as Equations (25)–(31).

(1). Trapezoidal distribution:

(25) $$\mu_{\tilde{D}}(\sigma_{i})=\{\begin{array}{ll} 0 & \sigma_{i}\in [0,\sigma_{F})\\ \frac{\sigma_{i}-\sigma_{F}}{\sigma_{0}-\sigma_{F}} & \sigma_{i}\in [\sigma_{F},\sigma_{0})\;\\ 1 & \sigma_{i}\in [\sigma_{0},+\infty ) \end{array}\;$$

(2). Quadratic parabola distribution:

(26) $$\mu_{\tilde{D}}(\sigma_{i})=\{\begin{array}{ll} 0 & \sigma_{i}\in [0,\sigma_{F})\\ {\left(\frac{\sigma_{i}-\sigma_{F}}{\sigma_{0}-\sigma_{F}}\right)}^{2} & \sigma_{i}\in [\sigma_{F},\sigma_{0})\;\\ 1 & \sigma_{i}\in [\sigma_{0},+\infty ) \end{array}\;$$

(3). Cubic parabola distribution:

(27) $$\mu_{\tilde{D}}(\sigma_{i})=\{\begin{array}{ll} 0 & \sigma_{i}\in [0,\sigma_{F})\\ {\left(\frac{\sigma_{i}-\sigma_{F}}{\sigma_{0}-\sigma_{F}}\right)}^{3} & \sigma_{i}\in [\sigma_{F},\sigma_{0})\;\\ 1 & \sigma_{i}\in [\sigma_{0},+\infty ) \end{array}\;$$

(4). Square root distribution:

(28) $$\mu_{\tilde{D}}(\sigma_{i})=\{\begin{array}{ll} 0 & \sigma_{i}\in [0,\sigma_{F})\\ {\left(\frac{\sigma_{i}-\sigma_{F}}{\sigma_{0}-\sigma_{F}}\right)}^{\frac{1}{2}} & \sigma_{i}\in [\sigma_{F},\sigma_{0})\;\\ 1 & \sigma_{i}\in [\sigma_{0},+\infty ) \end{array}\;$$

(5). Normal distribution:

(29) $$\mu_{\tilde{D}}(\sigma_{i})=\left\{ \begin{array}{ll} 0 & \sigma_{i}\in [0,\sigma_{F})\\ 1-e^{-{\left(\frac{\sigma_{i}-\sigma_{F}}{\sigma_{c}}\right)}^{2}} & \sigma_{i}\in [\sigma_{F},\sigma_{0})\;\\ 1 & \sigma_{i}\in [\sigma_{0},+\infty ) \end{array}\right.\;$$

(6). Γ distribution:

(30) $$\mu_{\tilde{D}}(\sigma_{i})=\{\begin{array}{ll} 0 & \sigma_{i}\in [0,\sigma_{F})\\ 1-e^{k(\sigma_{i}-\sigma_{F})} & \sigma_{i}\in [\sigma_{F},\sigma_{0})\;\\ 1 & \sigma_{i}\in [\sigma_{0},+\infty ) \end{array}\;$$

(7). Cauchy distribution:

(31) $$\mu_{\tilde{D}}(\sigma_{i})=\{\begin{array}{ll} 0 & \sigma_{i}\in [0,\sigma_{F})\\ \frac{1}{1+\alpha^{\prime }{\left(\sigma_{i}-\sigma_{F}\right)}^{-\beta^{\prime }}} & \sigma_{i}\in [\sigma_{F},\sigma_{0})\;\\ 1 & \sigma_{i}\in [\sigma_{0},+\infty ) \end{array}\;$$

Substituting the MFs into Equation (24), the fatigue life and damage parameters could be calculated. In Equations (25)–(31), *λ* = 0.75–0.95 is the lower boundary and other parameters are given as *σ*_c_ = 0.06*σ*_0_, *k* = 0.09, *α^’^* = 10, and *β^’^* = 2 [24,37,38].

**Figure 4:**
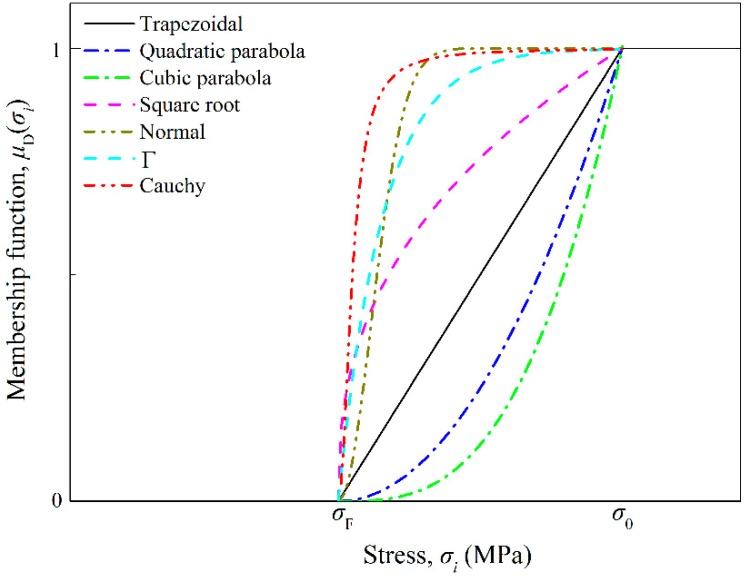
Curves of membership functions.

However, not all the above MFs are suitable for fatigue damage accumulation, applicable MFs should be selected for further study. The distributions of MFs in the stress range of 0 to *σ*_0_ are shown in Figure 4. The MFs can be sorted into three categories: (1) linear (the Trapezoidal MF), (2) concave (the Quadratic parabola MF and the Cubic parabola MF) and (3) convex (the Square root, the Normal, the Γ and the Cauchy MFs). The accumulated damage (Equation (24)) of the *i*th loading cycle consists of two parts: (1) the cumulative damage from the (*i* − 1)th cycle, and (2) the damage caused by the ith load. The total damage can be obtained by multiplying the MF, the strengthening coefficient and the corresponding damage. In this case, the damage accumulation rate caused by the low-amplitude loads is shrunk because the MF values from 0 to 1. Taking the linear MF as the reference, the concave MFs give lower membership values, which means the damage accumulated due to the same amplitude load would be smaller. It is why the Quadratic and the Cubic parabola MFs show higher life predictions than the experiment. For the convex MFs, the membership values are relatively large that leads to lower life predictions comparing with the trapezoidal MF. Therefore, the convex MFs would be more appropriate for the fatigue damage study.

## 3. Model Verification and Discussion

### 3.1. Model Verification

In this study, the uniaxial tensile-compression loading condition is considered and the variable amplitude load containing small loads is the main case. It has been stated that the low-amplitude stress could affect fatigue behaviour, so the extrapolated S–N curve is developed because the S–N curve is not applicable in this case [39]. Leipholz [40,41,42] also developed a modified S–N curve with Weibull distribution to consider low-amplitude loads. In order to validate the proposed model, fatigue lives of metallic specimen subjected to multi-level uniaxial loads are calculated using Wöhler model, Leipholz’s model, Chaboche model and the proposed model.

**Figure 5:**
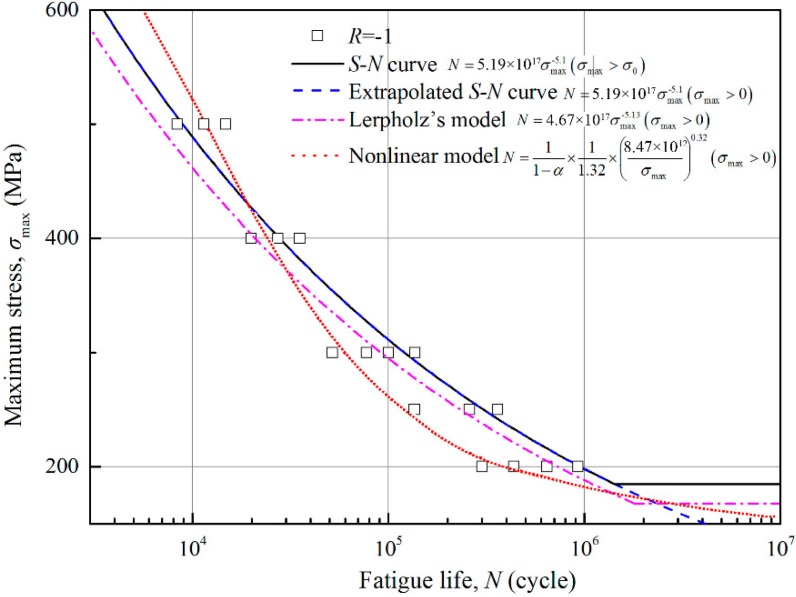
Fagitue model curves of 41Cr4 steel under *R* = −1.

In this section, four groups of experimental data of three different materials are employed to validate the proposed damage model. All experiments were conducted under multi-level loading condition including loading cycles that the amplitude is below the fatigue limit. The first case is experimental data of 41Cr4 steel [27] under multi-level symmetric loading condition, as listed in Table 1. Life predictions from different damage models are given in Figure 5 and Table 2. The parameters used are *σ*_0_ = 173.5 MPa and *m^’^* = 5.1 × 10^−7^. 

From Table 2, the Wöhler model (S–N curve) gives much higher prediction than the test, because all of the loading cycles below the fatigue limit are not considered to cause any damage, so the predicted life is so optimistic that unexpected failure would occur during the assumed safe period. In addition, the loading sequence effect is not considered as well. The Wöhler model (extrapolated S–N curve) and the Leipholz’s model consider the effect of the low-amplitude loads, but the improvements in calculation accuracy are limited because the effects of loading parameters on the damage accumulation are not taken into account. Furthermore, the predicted life of Leipholz’s model is lower than the life of Wöhler model (extrapolated S–N curve). The reason is that the Leipholz’s approach uses Weibull distribution, which is lower than the extrapolated S–N curve, for low-amplitude loading cycles. For the Chaboche model, it also shows a big gap between the prediction and the tested data—the predicted lives are far lower than they should be. It is because all the loading cycles under the fatigue limit are counted altogether, which leads to a faster damage accumulation process than the real situation. It further provides evidence that not all the low-amplitude loads can cause damage or strengthening effect. On the other hand, using the Chaboche model is safe for life prediction although some cost waste is inevitable. For the proposed model, the life predictions of the Trapezoidal, the Quadratic parabola and the Cubic parabola distribution MFs show large overestimations from the experimental results. But for the Square root, the Normal, the Γ and the Cauchy distribution MFs, the predicted lives are satisfying with considerable improvement based on the Chaboche model. Especially the Normal and the Cauchy distribution MFs show higher precisions with significantly reduction in relative errors (9% for the Normal distribution MF and 8% for the Cauchy distribution MF).

**Figure 6:**
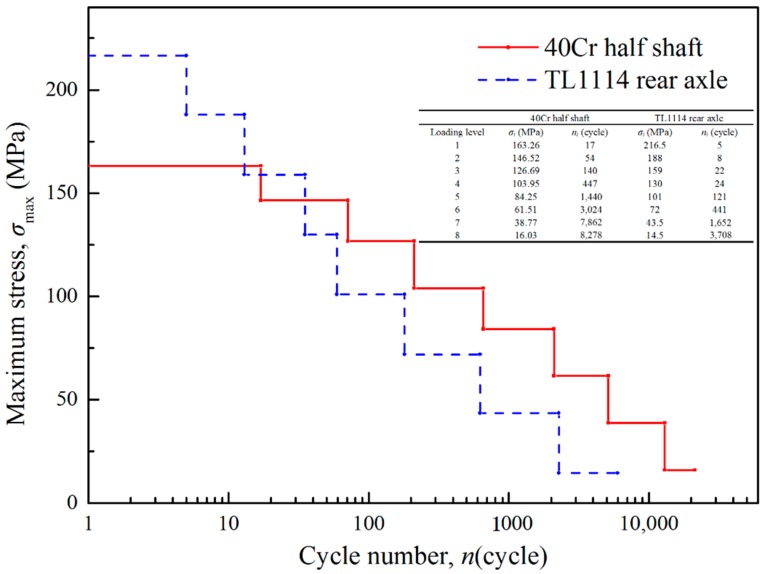
The loading histories of the 40Cr half shaft and the TL1114 rear axle.

The second case is the tested data of two engineering structures including a half shaft of 40Cr and a rear axle of TL1114 [43]. The fatigue limits of 40Cr and TL1114 are 101 MPa and 81 MPa respectively [44]. The loading histories of the half shaft and the rear axle are shown in Figure 6. It is found that the first 4-level loads are above the fatigue limit for the 40Cr half shaft, *σ*_5_ denotes the low-amplitude load with strengthening effect and the rest loads do not contribute to the fatigue damage accumulation. The first 5-level loads of the TL1114 rear axle are high-amplitude loads, *σ*_6_ is the low-amplitude load with strengthening effect, and *σ*_7_ and *σ*_8_ are neglected. Since this has been proved the most effective one among various MFs, only the proposed model with Cauchy MF is used in the second validation case. Under the above loading spectra of eight-level blocks, the average tested results are 1158 blocks for the half shaft and 490 blocks for the rear axle, respectively. The tested results and the predicted lives of the Chaboche model and the proposed model are listed in Table 3.

It is shown that the Chaboche model gives very safe life predictions: 21.5% and 14.9% lower than the tested data for the half shaft and the rear axle. As discussed above, this is because all the low-amplitude loads are considered in the Chaboche model as a whole no matter how small the amplitude is, so a higher damage accumulation rate results in a shorter fatigue life. In the proposed model, the contribution of each low-amplitude load to the fatigue damage is carefully identified. Some loading cycles are counted by strengthening the material and making damage at the same time, while some are neglected. As a result, the predicted life of the proposed model is very close to the tested data. The relative errors of life prediction are only 2.5% for the half shaft and 4.08% for the rear axle. It can be concluded that the proposed method with a selected MF (Cauchy) shows great agreements with the experimental data and the approach is robust for different materials and loading conditions.

### 3.2. Discussion

From the comparisons above, it can be seen that the Wöhler model (with S–N curve) provides the longest life prediction with the lowest accuracy, the Wöhler model (with extrapolated S–N curve) and the Leipholz’s model show better results but the predictions are still over-optimistic. It is partially because the nonlinearity of damage accumulation under different levels of load is not considered. The other reason is that the influence of the variation of load is not considered. Instead, the Chaboche model accounts the interaction between the loading parameters and the damage accumulation, and also considers the influences of the loading sequence and the load below the fatigue limit. So it is able to obtain better prediction than the first three linear models. However, the prediction of the Chaboche model is always too safe because it counts every loading cycle in damage accumulation regardless of amplitude. That is to say, some loading cycles with little contribution to damage are still calculated in the Chaboche model, which leads to a sort of waste in service. On this basis, the Chaboche model is modified by introducing a strengthening factor and a fuzzy membership function to deal with the low-amplitude loads. The fuzzy membership function is used to identify whether the loading cycle should be counted in damage accumulation by means of the amplitude of load. Then the strengthening factor is calculated and the extension effect of the low-amplitude load on fatigue life is added onto the damage accumulation. The modified model shows very close predictions to the experimental result. Sorting the low-amplitude loads into two classifications, the proposed method ensures the evaluation safety and reduces cost at the same time.

For the 41Cr4 steel case, the lower limit of the strengthening stress in Equations (25)–(31) is defined as *σ*_F_ = *λσ*_0_ (*λ* = 0.75) to ensure the safety of life prediction. It means nor material degradation or damage accumulation would occur if *σ_i_* < 0.75*σ*_0_ so the loads *σ*_8_ = 63 MPa in T1 and *σ*_7_ = 96 MPa and *σ*_8_ = 44 MPa in T2 are neglected in the computation. For the concave MFs, the Square root MF is the lowest in most stress range, so the corresponding life prediction is the highest, followed by the Γ MF. For the Normal and Cauchy MFs, the damage caused by the low-amplitude loads are considerable due to high membership values. Accordingly, the fatigue lives calculated by the Normal and Cauchy MFs are lower than others. Besides, the predicted life of the modified model depends on the function $\exp(m\sigma_{i+1})\times \mu_{\tilde{D}}(\sigma_{i+1})$. When $\exp(m\sigma_{i+1})\times \mu_{\tilde{D}}(\sigma_{i+1})<1$, the damage of low-amplitude loads is further reduced. In this case, a larger $\mu_{\tilde{D}}(\sigma_{i+1})$ brings a more accurate prediction. It is the reason that the relative error of convex MF is lower than that of the linear and concave MFs. On the contrary, when $\exp(m\sigma_{i+1})\times \mu_{\tilde{D}}(\sigma_{i+1})>1$, the damage of low-amplitude loads is enlarged. The predicted life is increasingly smaller than the experimental result with increasing $\mu_{\tilde{D}}(\sigma_{i+1})$. In summary, it is believed that, if *σ_i_* < *σ*_F_, the lower amplitude loads have so slight influence on damage accumulation that can be neglected. But the low-amplitude loads still cause the strengthening effect, which is consistent with the conclusion from Refs. [24,33].

**Figure 7:**
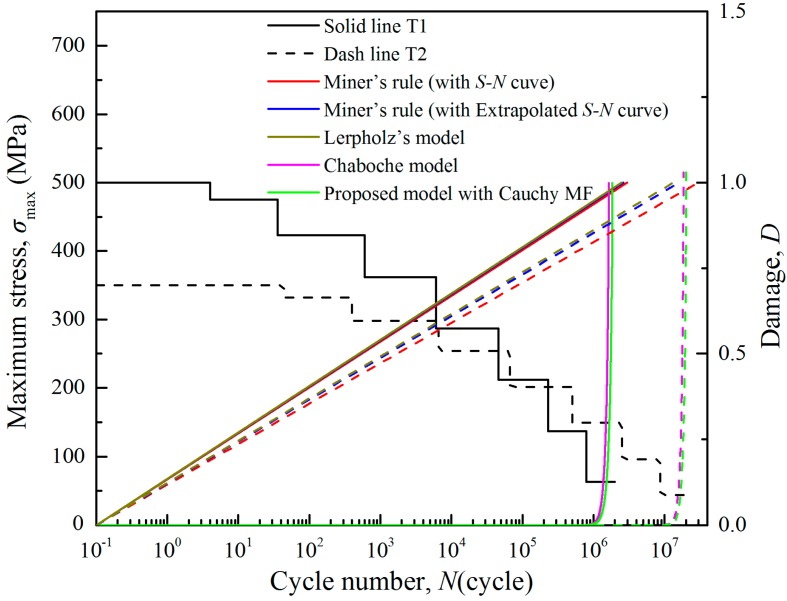
Comparisons in fatigue damage of 41Cr4 steel between the test data, the Wöhler model, the Chaboche model and the proposed model.

Figure 7 shows a comparison on the damage accumulation prediction between the Wöhler model (with S–N curve/extrapolated S–N curve), the Leipholz’s model, the Chaboche model and the proposed model. Firstly, the three linear damage models (two Wöhler models and the Leipholz’s model) give straight damage curves with constant rates, while the two nonlinear models show exponential curves with “low-high” damage accumulation rates. Although the Wöhler model with extrapolated S–N curve and the Leipholz’s model consider the low-amplitude load effect, the improvement in prediction accuracy is limited because the correlation between load parameter and damage is neglected. That is to say, for a damage model, the relationship between loading condition and damage accumulation rate is more important comparing with the low-amplitude effect. That is why the Chaboche model always can give better damage predictions than the linear models. In such nonlinear damage models, fatigue behaviour is regarded as a mutative process with varying damage accumulation rate. The cumulative damage is very small in the earlier stage of the fatigue development, grows along with the loading cycles, and then rapidly increases to a large value at the end. In this irreversible developing process, the low-amplitude effect becomes greater and greater as the damage accumulated. In the later stage of fatigue life, in particular, the impact of the low-amplitude loads on damage accumulation is quite considerable. On the other hand, not all the loads are worth consideration in the damage accumulation, especially for those low-amplitude loads far lower than the fatigue limit. If all loads are counted, such as the Chaboche model, excessive damage will be calculated that usually results in a much lower life prediction. Therefore, it is appreciated that low-amplitude load can be taken into account in the proposed model but with carefully identification to exclude redundant loading cycles.

## 4. Life Prediction of a Compressor Blade

An aeroengine compressor blade (5th in high pressure) is employed as a case study for the implementation of the proposed damage model. 

### 4.1. Blade Model

The finite element model of the blade consists of 734,995 nodes and 321,470 elements (20 node-hexahedrons/solid 186 for the body and 10-node tetrahedrons/solid 187 for the tenon). Taking the rotor axle as reference, the T-platform of the blade is constrained in the circumferential direction and the tenon is constrained in the normal direction of its side surface. The blade is made of Ti-6Al-4V alloy with density of 4440 kg/m^3^, Young’s modulus of 107 GPa, ultimate tensile strength of 1005 MPa and Poisson’s ratio of 0.34, respectively [17]. In our previous work [45], the fatigue limits of the blade were obtained approximately 345 MPa under *R* = −1 and 605 MPa under *R* = 0.1, respectively.

### 4.2. Numerical Simulation

**Figure 8:**
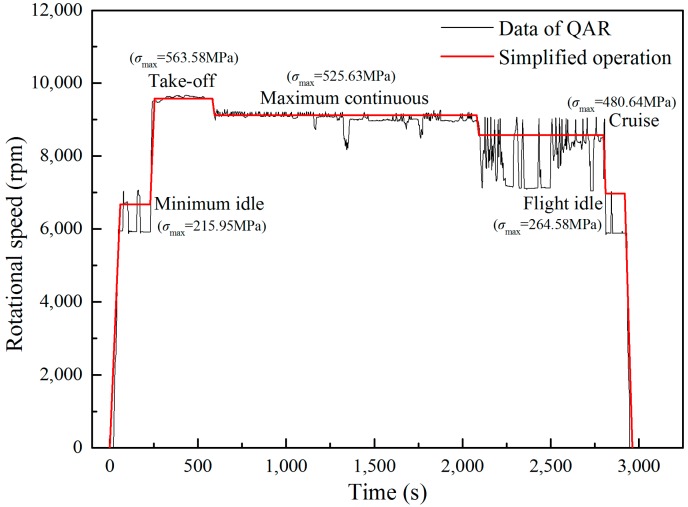
Typical load cases in a whole flight cycle.

In the stress analysis, two types of load are considered including the centrifugal force and the aerodynamic force [46]. The centrifugal force is a function of the rotational speed and the aerodynamic force is determined by a CFD simulation using ANSYS Workbench. The quick access recorder (QAR) data is used to obtain the speed spectrum of the blade and five typical load cases are obtained: minimum idle, take-off (maximum), maximum continuous, cruise, and flight idle, as shown in Figure 8. It is found that the stress concentrates at the blade root near the trailing edge on the suction side regardless of working condition. Although both aerodynamic and centrifugal forces are considered, the final stress condition of the dangerous point of the blade is considered as a tensile-compression case.

### 4.3. Results and Discussion

**Figure 9:**
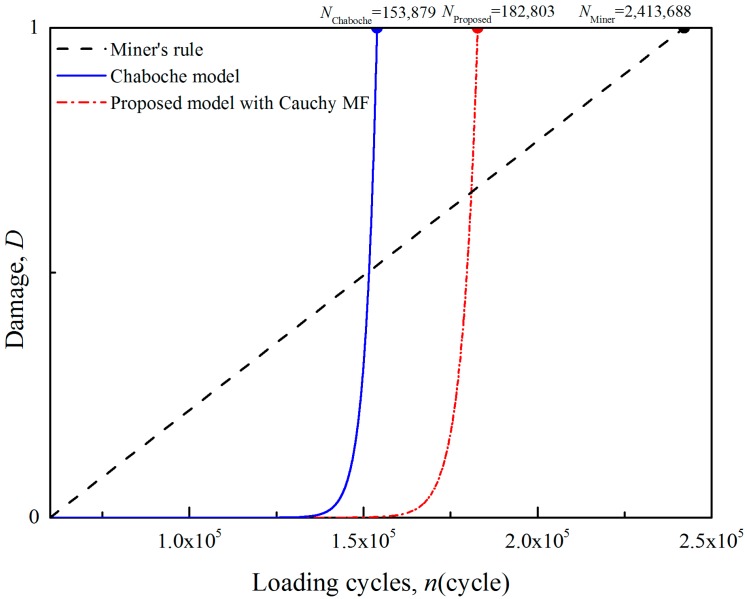
Damage predictions of blade using different models.

Based on the simulation, a stress history at the critical location of the blade is complied. The whole flight cycle is simplified into 6 sub-cycles: 0-take off-0 (L1), minimum idle-flight idle- minimum idle (L2), minimum idle-take off-minimum idle (L3), cruise-maximum continuous-cruise (L4), flight idle-maximum continuous-flight idle (L5) and 0-minimum idle-0 (L6). The sub-cycles L1, L3 and L5 are high-amplitude loading cycles and L2, L4 and L6 are low-amplitude loading cycles. Then the fatigue damage of the blade is analysed by the Wöhler model, the Chaboche model and proposed model with Cauchy MF, as shown in Figure 9. 

As expected, the Wöhler’s result is the highest one (2,413,688 cycles), the Chaboche’s prediction is the lowest (153,879 cycles) and the proposed model gives a moderate result (182,803 cycles). In terms of damage, the accumulation rates of the two nonlinear models are equally small for the first 125,000 cycles. Although the Chaboche model counts all the low-amplitude loads but the proposed model does not, the strengthening effect and the damage caused by the low-amplitude load are very slight in the early stage of fatigue with low initial damage. So, the two nonlinear models show similar predictions. Beyond 125,000 cycles, an obvious deviation can be found between the two nonlinear models due to the difference in counting mechanism of low-amplitude load. The damage accumulation of the proposed model becomes slower because some of the low-amplitude loads are neglected and the strengthening effect is added. 

## 5. Conclusions

(1) Low-amplitude load below the fatigue limit still contributes to the damage accumulation but the contribution depends on the damage existed. The effect of the low-amplitude load on damage accumulation increases with increasing initial damage.

(2) Although the low-amplitude load below fatigue limit should be counted on the damage accumulation, further identification on the load amplitude is needed. The strengthening effect can be considered through an exponential function and the damage can be calculated by a fuzzy membership function.

(3) The proposed Cauchy MF based damage model has the most satisfying precision in life prediction, which can consider the low-amplitude load effect properly to extend the predicted life to some extent, but the evaluation is still conservative to ensure the engineering safety.

## Figures and Tables

**Table 1:** The experimental data of 41Cr4 steel [27].

T1			T2		
Loading Level	*σ_i_* (MPa)	*n_i_* (Cycle)	Loading Level	*σ_i_* (MPa)	*n_i_* (cycle)
1	500	4	1	350	44
2	475	32	2	332	352
3	423	560	3	298	6160
4	362	5440	4	254	59,840
5	287	40,000	5	201	440,000
6	212	184,000	6	149	2,024,000
7	137	560,000	7	96	6,160,000
8	63	1,210,000	8	44	13,310,000

**Table 2:** Comparisons between different fatigue models and test.

Method	T1		T2	
	Fatigue Life (Cycle)	Relative Error (%)	Fatigue Life (Cycle)	Relative Error (%)
Test	2,000,036	-	22,000,396	-
Wöhler model (S–N curve)	2,980,258	49.01	29,739,204	35.18
Wöhler model (extrapolated S–N curve)	2,632,432	31.62	16,045,967	27.07
Leipholz’s model	2,575,804	28.79	14,309,047	34.96
Chaboche model	1,639,758	18.02	18,460,752	16.09
Modified model (Trapezoidal MF)	2,247,234	12.35	24,601,638	11.82
Modified model (Quadratic parabola MF)	2,320,038	15.99	25,115,005	14.16
Modified model (Cubic parabola MF)	2,387,111	19.35	26,078,819	18.54
Modified model (Square root MF)	2,208,623	10.43	24,161,781	9.83
Modified model (Normal MF)	1,819,343	9.03	20,027,736	8.97
Modified model (Γ MF)	2,185,874	9.29	24,115,718	9.61
Modified model (Cauchy MF)	1,842,027	7.96	20,156,752	8.38

**Table 3:** Comparisons on fatigue lives between damage models and test.

Method	40Cr Half Shaft		TL1114 Rear Axle	
	Fatigue Life (Load Blocks)	Relative Error (%)	Fatigue Life (Load Blocks)	Relative Error (%)
Test	1158	-	490	-
Chaboche model	909	21.5	417	14.9
Modified model (Cauchy MF)	1129	2.5	510	4.08

## Nomenclature

*D*	fatigue damage
*D_i_*	cumulative damage at the *i*th loading cycle
*D*_0_	initial damage
*D*_c_	critical damage
*m^′^*	strengthening coefficient
*N*	loading cycles
*n_i_*	loading cycles of the *i*th-level load
*N*_f_	fatigue life
*N_i_* ^*^	equivalent fatigue life subjected to the *i*th-level load
*Y_i_*	cyclic ratio
$\mu_{\tilde{D}}(\sigma_{i})$	membership function
*Σ*	stress
*σ_i_*	stress of the *i*th-level load
*σ*_a_	stress amplitude
*σ*_F_	lower limit of strengthening stress
*σ*_m_	mean stress
*σ*_max_	maximum stress
*σ*_min_	minimum stress
*σ*_0_	fatigue limit
*b*_0_, *M*_0_, *α*, *β*	material parameters
*k*, *α^′^*, *β^′^*, *σ*_c_	membership function parameters
